# Supplementary material for: Human surfactant protein A inhibits SARS-CoV-2 infectivity and alleviates lung injury in a mouse infection model
Source: Front Immunol. 2024 Mar 26;15:1370511. doi: 10.3389/fimmu.2024.1370511 (PMC11002091; doi:10.3389/fimmu.2024.1370511)
Supplement: Supplementary file 1 [file DataSheet_1.pdf]

Human surfactant protein A inhibits SARS-CoV-2 infectivity and alleviates lung injury in a mouse infection model

Ikechukwu B Jacob[1,2]; Amanda Gemmiti[3]; Weichuan Xiong[1], Erin Reynolds[2]; Brian, Nicholas [3], Saravanan Thangamani[2]; Hongpeng Jia [4]; Guirong Wang [1,2,*]

[1]Department of Surgery, the State University of New York (SUNY) Upstate Medical University, Syracuse, NY, 13210, USA [2]Department of Microbiology & Immunology, SUNY Upstate Medical University, Syracuse, NY, 13210, USA

[3]Department of Otolaryngology, SUNY Upstate Medical University, Syracuse, NY, 13210, USA

[4]Department of Surgery, Johns Hopkins University, Baltimore, Maryland

Running title: Role of SP-A against SARS-CoV-2 infectivity

<u>Corresponding author:</u>
Guirong Wang, PhD (Dr. rer. Nat.)
Department of Surgery, UH Room 8715
SUNY Upstate Medical University
750 E Adams St,
Syracuse, NY, 13210, USA
Phone: 315-464-6283
Fax: 315-464-6236
E-mail: wangg@upstate.edu

SUPPLEMENTARY INFORMATION

Materials and Methods

Human SP-A Protein

Native human SP-A (hSP-A) was isolated and purified from bronchoalveolar lavage fluid (BALF) of alveolar proteinosis patients as described previously (1). The purity of the SP-A preparation was confirmed by SDS-PAGE followed by silver staining and then filtered through a 0.2-micron filter to remove potential contaminants.

Mouse Infection and Sample Processing

SP-A hTG mice, including hACE2/SP-A KO and hACE2/mSP-A (K18) mice were anesthetized using isoflurane and infected intranasally (i.n.) with 20 µl (10 µl/nose) of virus suspension containing 1×10^3 PFU of SARS-CoV-2 (delta variant) in 1X MEM. Control (SHAM) mice were inoculated with 1X MEM. Mice were sacrificed by anesthesia and exsanguination at 6 dpi to obtain lung samples for viral load analysis plaque assay.

Tissue Collection

Mock and SARS-CoV-2 infected mice (3 groups: hACE2/SP-A1 ($6A^2$), hACE2/mSP-A and hACE2/SP-A KO) were anesthetized with isoflurane. Mouse lungs (n= 7/group) were harvested and frozen immediately at -80 ^oC for further processing. The lungs were weighed and homogenized in cold PBS and then centrifuged at 21130 RCF for 5 mins and supernatants were collected for viral load analysis using plaque assay.

Competition Assays

SP-A (10 µg/ml) was incubated simultaneously in a buffer containing 10 mM each of increasing concentrations of maltose for 1 h in plates previously coated with either S protein (1 µg/ml) or RBD (50 ng/ml, VANC00B, R&D Systems). As a control, SP-A was incubated in a 5 mM $CaCl_2$-containing buffer without sugars. In another experiment, SP-A (10 µg/ml) was simultaneously incubated with increasing mannose concentrations in wells previously immobilized with 0.2 µg/ml biotinylated ACE2. Bound biotinylated ACE2 and SP-A was detected by incubating with Streptavidin-HRP (VANC00B, R&D Systems) following incubation with SP-A polyclonal antibody (1:1000) for 1 h at room temperature and developed as described above. All analyses were carried out in duplicate (n=3).

Plaque Assay

The potential antiviral activity of human SP-A against SARS-CoV-2 infectivity *in vivo* was assessed by plaque assay in Vero E6 cells. Viral titer was quantified using supernatants from SARS-CoV-2 infected lung homogenates. Briefly, Confluent monolayers of Vero E6 in 24-well plates were infected with 10-fold serial dilutions of supernatants from each infected mouse group. The cells were incubated for 1 h with intermittent rocking at 37^oC. The unbound virus was removed and overlayed with 2% methylcellulose and cultured for another 72 h. Upon the development of plaques, cells were fixed with 10% formalin for 1 h and stained with 0.05% (w/v) crystal violet in 20% methanol and plaques were counted.

Statistical analysis

All experimental data are presented as mean ± standard error and statistically analyzed using GraphPad Prism 8.0 (GraphPad Software, San Diego, CA, USA). Comparisons between two independent groups were performed using Student's *t*-test or multiple groups using one-way ANOVA. It was considered statistically significant when P<0.05.

Results

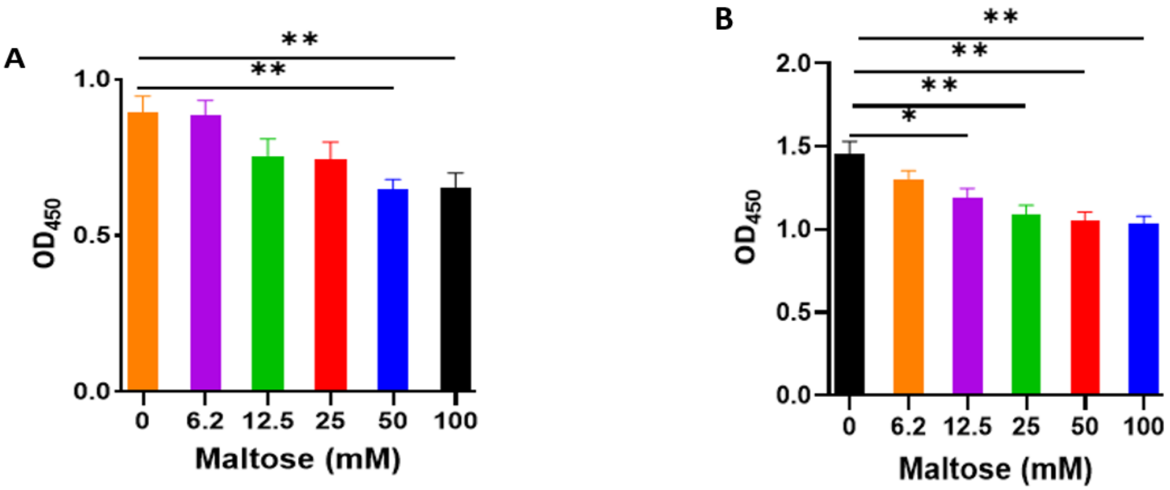

Supplement Figure E1: Increasing concentrations of Maltose were unable to completely inhibit SP-A interaction with S protein (A) and RBD (B). This suggests that domains other than CRD of SP-A may also interact with SARS-CoV-2 S protein and RBD. The experiments were carried out in triplicates (n=3). The error bars represent SEM and *P<0.05; **P<0.01 equals the level of significance when compared with control (0 mM) samples using unpaired t-test analysis

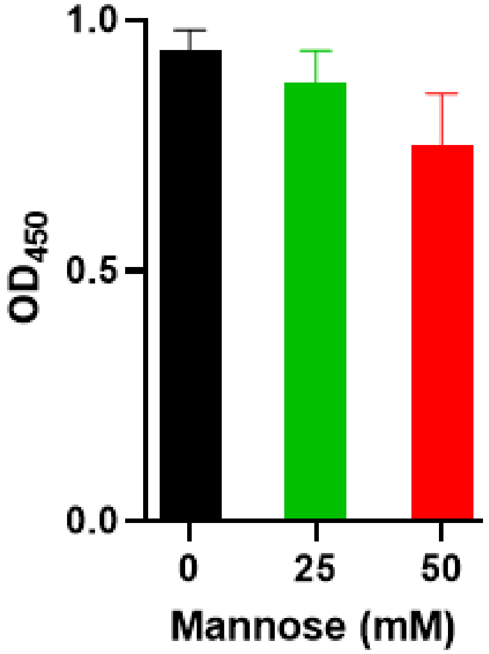

Supplement Figure E2: Mannose (a preferred ligand for SP-A) weakly competitively inhibited SP-A interaction with human ACE2. Biotinylated ACE2-coated plates (0.2 µg/ml) were incubated with SP-A at 10 µg/ml in the presence of 25 mM and 50 mM mannose. Control samples were incubated with SP-A in 5 mM $CaCl_2$ buffer without sugars (0 mM). The plates were developed as described in the Methods section. The experiments were carried out in duplicate (n=3).

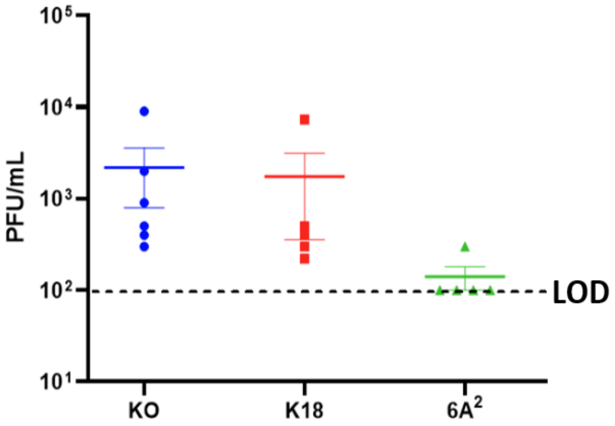

Supplementary Figure E3. Human SP-A Attenuates SARS-CoV-2 Load in Mice. Viral load in the lungs of SARS-CoV-2-infected mice was examined by plaque assay of lung homogenates from the infected mouse groups on Vero cells. The horizontal dotted line shows the limit of detection (LOD). The data indicate undetectable levels of infectious virus in mice carrying the hSP-A1 gene ($6A^2$) compared to K18 and SP-A KO mice.

References:

1. Wang G, Phelps DS, Umstead TM, Floros J. Human SP-A protein variants derived from one or both genes stimulate TNF-α production in the THP-1 cell line. *American Journal of Physiology - Lung Cellular and Molecular Physiology* 2000; 278: 946-954.
